# Rigorous treatment of pairwise and many-body electrostatic interactions among dielectric spheres at the Debye–Hückel level

**DOI:** 10.1140/epje/s10189-021-00131-9

**Published:** 2021-10-18

**Authors:** O. I. Obolensky, T. P. Doerr, Yi-Kuo Yu

**Affiliations:** 1 National Center for Biotechnology Information, National Library of Medicine, National Institutes of Health, Bethesda, MD 20894 USA; 2 The A.F. Ioffe Institute, St. Petersburg, Russia

## Abstract

**Abstract:**

Electrostatic interactions among colloidal particles are often described using the venerable (two-particle) Derjaguin–Landau–Verwey–Overbeek (DLVO) approximation and its various modifications. However, until the recent development of a many-body theory exact at the Debye–Hückel level (Yu in Phys Rev E 102:052404, 2020), it was difficult to assess the errors of such approximations and impossible to assess the role of many-body effects. By applying the exact Debye–Hückel level theory, we quantify the errors inherent to DLVO and the additional errors associated with replacing many-particle interactions by the sum of pairwise interactions (even when the latter are calculated exactly). In particular, we show that: (1) the DLVO approximation does not provide sufficient accuracy at shorter distances, especially when there is an asymmetry in charges and/or sizes of interacting dielectric spheres; (2) the pairwise approximation leads to significant errors at shorter distances and at large and moderate Debye lengths and also gets worse with increasing asymmetry in the size of the spheres or magnitude or placement of the charges. We also demonstrate that asymmetric dielectric screening, i.e., the enhanced repulsion between charged dielectric bodies immersed in media with high dielectric constant, is preserved in the presence of free ions in the medium.

**Graphic abstract:**

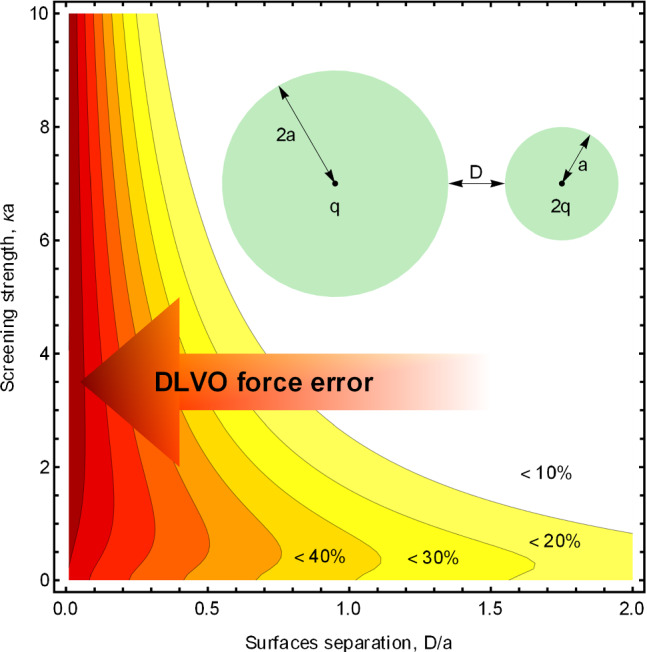

## Introduction

The search for methods providing more accurate descriptions and deeper understanding of electrostatic interactions among charged dielectric spheres in ionic solutions remains, despite its long history, a subject of high interest, as proven by a persistent stream of theoretical and experimental publications. This continued interest is motivated by new types of dielectric objects that could be approximated by dielectric spheres and whose properties and interactions are important for other fields of physics, chemistry, materials design and biology.

In order to describe charged dielectric spheres in ionic solutions, it is desirable to choose models for the charged dielectric spheres and the electrolyte solution that may be productively combined, solved consistently, and, ideally, yield a rigorous solution. The most straightforward formulation for the interaction of the charged dielectric spheres is a traditional differential equation boundary value problem of electrostatics. Thus, one has to introduce a compatible model (differential equation) for describing the ionic solution.

It is commonly accepted that, under not too extreme circumstances, each ion in the ionic solution can be considered freely diffusing through the solution in a canonically averaged electric potential $\phi (\mathbf{r})$, created by an “atmosphere” of other free ions and ionized colloidal particles. On top of that, the ions are assumed to be thermalized according to Maxwell–Boltzmann statistics. These assumptions give rise to the Poisson–Boltzmann equation:

1 $$\begin{aligned} \nabla ^2 \phi (\mathbf{r}) = -\frac{4 \pi }{\epsilon _o} \sum _s c_s\, q_s \,e^{-\beta \, q_s \, \phi (\mathbf{r})}, \end{aligned}$$

where $q_s$ and $c_s$ are the charge and average concentration of the ions of species *s*, $\epsilon _o$ is the dielectric function of the solution, and $\beta$ is the inverse temperature. It is worth mentioning that the solution of the Poisson–Boltzmann equation, while certainly being useful for providing physical insights, cannot be held as a gold standard of colloidal electrostatics. Indeed, as was originally pointed out by Onsager [1] and Fowler [2], the solutions of the Poisson–Boltzmann equation violate a general reciprocity principle, according to which the work done for bringing an ion *j* to its position $\mathbf{r}_j$ in the presence of ion *k* at position $\mathbf{r}_k$ must be equal to the work done for bringing the ion *k* to position $\mathbf{r}_k$ in the presence of ion *j* at position $\mathbf{r}_j$ [3–6] (see a brief discussion of this in Appendix A).

If the average potential $\phi (\mathbf{r})$ is sufficiently small throughout the solution, $\beta \, q_s\, \phi (\mathbf{r}) \ll 1$, the exponential in Eq. (1) can be expanded into a power series and only the first-order term kept, as was originally done by Debye and Hückel [7] (the zeroth-order term vanishes due to the overall charge neutrality):

2 $$\begin{aligned} \nabla ^2 \phi (\mathbf{r}) = \kappa ^2 \phi (\mathbf{r}), \end{aligned}$$

where

3 $$\begin{aligned} \kappa = \sqrt{4\pi \frac{\beta }{\epsilon _o} \sum _s c_s q_s^2} \end{aligned}$$

is the inverse Debye screening length. Using the Debye–Hückel equation (2) instead of the Poisson–Boltzmann equation (1) may in fact be more justified physically as, in the words of Lars Onsager, “... as soon as the higher terms in the Poisson–Boltzmann equation become important, we can no longer expect the ionic atmospheres to be additive, and then the Poisson–Boltzmann equation itself becomes unreliable.” [3]

Importantly, not only does the solution of the Debye–Hückel equation satisfy the reciprocity principle, it can further be shown [5] that it is the exact small $\kappa$ limiting form of the Poisson equation for the canonically averaged potential. Thus, curiously, the Debye–Hückel equation has in some ways a firmer physical foundation than the Poisson–Boltzmann equation, in spite of the fact that the former is obtained as a linearization of the latter. Moreover, the range of validity and utility of the Debye–Hückel equation may be expanded by choosing $\kappa$ appropriately [8–11]. Since the Debye–Huckel equation obeys certain symmetries violated by the Poisson–Boltzmann equation, allows for rigorous solution, and can have its range of applicability extended by considering $\kappa$ to be an effective parameter, the Debye–Hückel theory seems a desirable choice for combining with a theory of charged dielectric spheres. There are field theoretical [12] and integral equation [13] techniques for investigating the thermodynamics of ionic solutions that can potentially address the nonlinear regime. However, as we are attempting to combine an electrolyte theory with an electrostatic boundary value problem, and we further anticipate application for simulations, such methods do not seem appropriate for the purposes considered here.

However, one needs to be careful to verify that the Debye–Hückel linearization is valid. In order to do so, one needs to actually solve the electrostatics problem and find the maximum magnitude of the potential, making sure that it is smaller than $1/\beta = k_\mathrm{B} T$ ($\approx 26$ meV for a solution at 298 K).

**Fig. 1:**
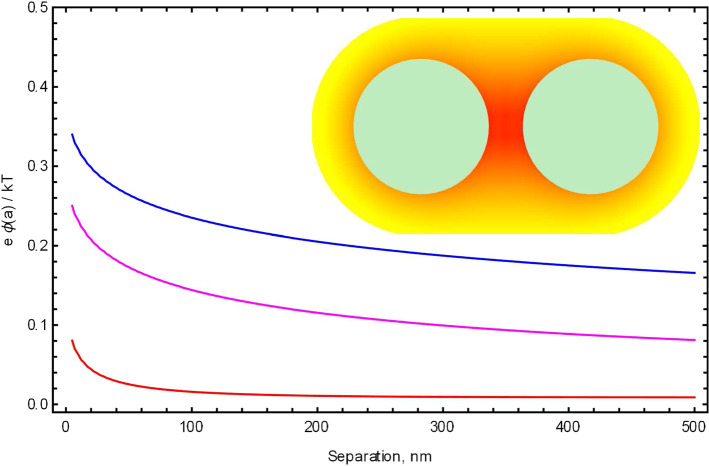
The electrostatic energy $e\, \phi (r)$ of an ion in an ionic solution for interaction of two polystyrene particles of radius $a=1\,\upmu$m, uniformly charged to $10^5$ elementary charges *e*, calculated near the surface of one of the spheres ($r=a$) and measured in units of $k_\mathrm{B}T$. The ratio $e \phi (a) / k_\mathrm{B}T$ is plotted as a function of separation between the spheres’ surfaces for three screening strengths: $\kappa a = 0.1$ (upper, blue line), $\kappa a = 1$ (medium, magenta line), $\kappa a = 10$ (bottom, red line). The dielectric constant of polystyrene is taken to be 2.6, while for the solvent $\epsilon _o$ is taken equal to 78.3. The potential is calculated using the rigorous formalism presented in Sect. 2. Note that condition $e \phi (a) / k_\mathrm{B}T \ll 1$ is the applicability condition for the Debye–Hückel equation. In the inset, we provide a two-dimensional cross-sectional view of the strength of the potential for the spheres separated by 250 nm, which ranges from approximately 0.11 $k_\mathrm{B}T$ (red background) to 0.02 $k_\mathrm{B}T$ (yellow background)

In Fig. 1, we plot the electrostatic energy $e\, \phi (r)$ of an ion in an ionic “atmosphere”, in the units of $k_\mathrm{B} T$, as a function of the distance between the surfaces of two spheres. The potential is obtained as the rigorous solution at the Debye–Hückel level (see Sect. 2) and is calculated at a point where it attains its maximum value, at the surface of one of the spheres, and at the point closest to the other sphere. The parameters of this two-sphere system were chosen to be similar to conditions used in a set of optical tweezer experiments that measured the interaction force between two charged colloidal particles [14]. We see from the figure that for polystyrene particles of radius $a=1\,\upmu$m, uniformly charged to $10^5$ elementary charges *e*, the Debye–Hückel linearization is valid up to the point where the spheres touch.

The Debye–Hückel linearization is generally less accurate for weaker solutions (smaller screening strength $\kappa a$), as it is clearly seen in Fig. 1. For demonstration purposes, the smallest $\kappa a$ we used in Fig. 1 was 0.1, while the molarities used in the experiment [14] would result in $\kappa a$ ranging in tens and hundreds. Therefore, the Debye–Hückel equation would still be applicable even if the colloidal particles were even more highly charged.

Another important conclusion that can be drawn from Fig. 1 is that calculating potential at the surface of an isolated sphere in an ionic solution may not be sufficient for judging the validity of the Debye–Hückel linearization. Indeed, the potential is greatly enhanced when two spheres interact with each other. The potential can increase several fold when two charged spheres are in a close proximity, due to significant values of induced surface charge. This circumstance makes finding an accurate solution to the electrostatic problem even more crucial.

Soon after Debye and Hückel proposed their linearized Poisson–Boltzmann model for describing properties of electrolytes [7], the question of how to correctly describe within this model the interaction between charged dielectric bodies was actively debated [15–19]. It was unclear what role the van der Waals interaction plays as compared to the direct electrostatic interaction and even whether electrostatic interaction between similarly charged particles is repulsive or attractive due to the presence of free ions in the solution [20, 21].

The consensus model was formulated by Derjaguin and Landau [22, 23] and independently by Verwey and Overbeek [24], colloquially joined together and known as the DLVO theory [25]. The DLVO approximation (with or without the *ad hoc* van der Waals term) is widely used when a quick estimate of interaction energy or force is needed [26]. While the DLVO energy is beautiful in its simplicity and physical clarity and remains in use (see, for a recent example, Ref. [27]), its numerical accuracy and the range of phenomena it can capture are inherently limited.

Beyond the DLVO approximation, there exists a very wide variety of theoretical approaches to colloidal particles in an ionic solution. Not aiming at giving a comprehensive review of these efforts and focusing primarily (but not exclusively) on studies considering dielectric spheres in electrolyte solutions, we can refer the reader to some recent works in the field, employing the method of image charges [28], perturbative methods [29], integral equation methods [30], expansions of induced surface charges into a series of polynomials [31], solving the Poisson equations in specialized coordinate systems [32], discretization of (potentially arbitrary) dielectric surfaces aimed at finding numerical solutions for arbitrary geometries [33, 34], direct numerical solution of differential equations [35, 36], and so-called hybrid methods [37]. There are, of course, various additional issues such as considering the finite size of the electrolyte ions [38, 39] or developing an analytic solution for interaction of spheroidal objects [40], but these are farther afield for the purposes of this article. A number of methods have been developed to describe systems with higher multipole moments. For example, Hoffman et al. [41] considered higher multipoles for a Debye–Hückel system, but only for a single sphere. Also, Boon et al. [42] and de Graaf et al. [43] considered higher multipole moments at the Poisson–Boltzmann level for a single sphere with axially symmetric surface charge. Making progress on the issue of many-body effects, Russ et al. [44] investigated few body forces but were restricted to considering monopole charges and to low order in the number of spheres.

Among the methods using the Debye–Hückel theory and employing an expansion of surface charge into a polynomial series and using the boundary conditions to find the expansion coefficients of such series, we should highlight the rigorous formalism of Fisher et al. [9]. They devised a set of polynomials to serve as the basis set of the expansion and were able to solve the boundary value problem for two identical spheres in an electrolyte solution with either like or opposite point charges at their centers, thereby obtaining an expression for the interaction energy. Even though a direct analytic comparison would be cumbersome, their result for this symmetric two-sphere system is numerically equivalent to that obtained within the general formalism developed recently [39] (even the convergence rate is the same, see Fig. 9 later).

Sushkin and Phillies [45] attempted to address the general case of two arbitrary spheres with an arbitrary collection of point charges within each. Their formalism is physically sound, but suffers from unpolished presentation, calling for a great deal of further simplification and clarification. Moreover, the printed manuscript probably contains typos.^1^

During the recent decade, Besley and Stace et al. have achieved significant progress in various aspects of interactions between two dielectric bodies in vacuum and in an electrolyte medium, reported in a series of papers [40, 46–51]. Their formalism [46], although imposing azimuthal symmetry with respect to the axis connecting the centers of the two spheres, was demonstrated to provide useful insights, such as dependence of attraction between two spheres with same charge immersed in a medium of lower dielectric constant on asymmetry of charge and radii of the interacting spheres [47, 50]. Their analysis uses a re-expansion of modified Bessel functions about a new center to allow matching of the boundary conditions at the surface of each sphere. However, multiple re-expansions are required in order to obtain the necessary Legendre polynomials, making controlling the numerical accuracy somewhat difficult since rather than a single maximum multipole $\ell _\mathrm{max}$, the multiple re-expansions introduce additional sums that need to be cut off in a consistent manner.

In a series of efforts [52–55], we have undertaken the development of a rigorous method for determining to any desirable precision the interaction energies for an arbitrary number of dielectric spheres in a dielectric medium. The most recent work [39] contains a general formalism, rigorous within the Debye–Hückel approximation, for describing interactions among dielectric spheres immersed in an electrolyte solution. The only other surface charge method of a similar generality and rigor that we are aware of is due to Lotan and Head-Gordon [56]. The downside of their method, however, is that transformations of coordinates when re-expanding at different locations are handled via an iterative numerical procedure. This significantly complicates computations, reduces their efficiency, and, even more importantly, introduces another source of uncertainty in the accuracy of the numerical results on top of cutting off polynomial expansions of the surface charge distributions.

In this article, we apply the general formalism [39] to detailed analysis of interactions in the case of two, three and four spheres. A terse summary of the general formalism may be found in Appendix B. We work out the simplifications that are possible for the particular case of two spheres and even further simplifications for the case of two spheres with axially symmetric charge distributions. In the latter case, it is possible to derive a simple expression for the force in terms of the same set of linear equations as for the energy. The significant new physical results are as follows. We demonstrate in Sect. 2.1 that asymmetric dielectric screening [52, 57], i.e., the enhanced repulsion between charged dielectric bodies immersed in media with high dielectric constant, known to occur in the absence of ions, is preserved in the presence of free ions in the medium. In Sect. 3, we compare the baseline DLVO approximation [25] to the results of the rigorous formalism. Indeed, only by comparing to the exact solution of the Debye–Hückel problem can the accuracy of DLVO theory (or any of its proposed modifications or replacements) be correctly and reliably assessed. We highlight the circumstances under which one can expect larger errors for the DLVO theory. In the last section, we discuss the accuracy of approximating the full interaction energy as a sum of pairwise interactions using examples of three- and four-sphere systems. Finally, we illustrate how the speed of convergence depends on variation of radii, charge magnitude and charge placement in the system.

## Two spheres

In order to represent charged colloidal particles in an ionic solution, we consider a system of dielectric spheres in a medium (water) with dielectric constant $\epsilon _o$ containing freely diffusing, thermalized ions. Sphere *i* has dielectric constant $\epsilon _i$, radius $a_i$, position $\mathbf{r}_i$, and free charge that can be represented by standard electric multipole moments $q_{lm}$ ($l=0,1, \ldots ,\infty$ and $m=-l,-l+1, \ldots ,l$) or equivalently [39, 54] by surface moments $\overline{Q}_{lm}$. This free charge distribution is fixed (we do not consider a dynamic chemical charge regulation scheme for the free charge), but there will be additional induced charge at the interface between the regions of differing dielectric constant. The spheres are embedded in an electrolyte solution that will be modeled by the Debye–Hückel theory and whose properties are therefore summarized by the inverse Debye screening length $\kappa$. We will, for the most part, regard $\kappa$ as a parameter. Thus, Eq. (3) will not be binding.

Within the framework of the general formalism for this system developed in Ref. [39] and briefly recapitulated in Appendix B, the distribution of the fixed (also called free) charge $\rho (\mathbf {s})$ at each sphere is expanded into a series using the basis set of spherical harmonics $Y_{\ell m}$. The (yet unknown) induced charge distribution at each sphere is also expanded with the same basis set, and the boundary conditions on the potential at the surface of each sphere then lead to a system of linear equations. The variables in this system are scaled spherical components of the net (free + induced) charge distribution $\breve{Q}_{\ell m}^{+}$. Having determined these components by solving the system of equations, one can subsequently find the induced charge distributions on each sphere, the electric potential at any point of space, as well as the electrostatic interaction energy.

### Two spheres with arbitrary charge distributions

For a system of only two spheres, the general system of linear equations simplifies not only due to fewer expansion centers, but also due to the fact that for two spheres there is only one vector connecting the centers of the spheres, and this vector $\mathbf {L} \equiv \mathbf {L}_{1\rightarrow 2} = - \mathbf {L}_{2\rightarrow 1}$ can always be directed along the *z*-axis. The spherical harmonics depending on the orientation of this vector are, therefore, only nonzero for zero momentum projections:

4 $$\begin{aligned} Y_{\ell _2 m_2} \left( \hat{L}_{1\rightarrow 2}\right)= & {} \sqrt{\frac{2\ell _2+1}{4\pi }} \delta _{m_2 0},\nonumber \\ Y_{\ell _2 m_2} \left( \hat{L}_{2\rightarrow 1}\right)= & {} (-1)^{\ell _2} \sqrt{\frac{2\ell _2+1}{4\pi }} \delta _{m_2 0}. \end{aligned}$$

As small as it seems, this circumstance allows one to derive a quite simple, yet still rigorous, system of equations for solving the electrostatics problem of two spheres in an ionic solution.

With (4) in mind, the general system of equations (39) can be reduced to:

5 $$\begin{aligned} \overline{Q}^1_{\ell m}&\!=&i_\ell \left( \kappa a_1\right) \, k_\ell \left( \kappa a_1\right) \, \mathcal {K}\left( \ell , \kappa a_1\right) \, \breve{Q}^{1+}_{\ell m} \nonumber \\&+ \sum _{\ell _1} (-1)^{\ell _1}\, i_\ell (\kappa a_1) \, i_{\ell _1}\left( \kappa a_2\right) \, \mathcal {I}\left( \ell , \kappa a_1\right) \, \mathcal {H}_{\ell m \ell _1} (\kappa L) \; \breve{Q}^{2+}_{\ell _1 m} \nonumber \\ \overline{Q}^2_{\ell m}= & {} i_\ell \left( \kappa a_2\right) \, k_\ell \left( \kappa a_2\right) \, \mathcal {K}\left( \ell , \kappa a_2\right) \, \breve{Q}^{2+}_{\ell m} \nonumber \\&+ \sum _{\ell _1} (-1)^{\ell }\, i_\ell \left( \kappa a_2\right) \, i_{\ell _1}\left( \kappa a_1\right) \, \mathcal {I}\left( \ell , \kappa a_2\right) \, \mathcal {H}_{\ell m \ell _1} (\kappa L) \breve{Q}^{1+}_{\ell _1 m}.\nonumber \\ \end{aligned}$$

Here for convenience we use explicit enumeration of the spheres. The source terms in (5) are the spherical components of the free charge distribution (surface moments) $\overline{Q}_{\ell m}$,

6 $$\begin{aligned} \overline{Q}_{\ell m} \equiv \sqrt{4\pi }\, \frac{q_{\,\ell m}}{a^\ell } = \frac{\sqrt{4\pi }}{a^\ell } \int \rho (\mathbf {s}\,) \; s^\ell \, Y_{\ell m}^*(\hat{s}) \, d\mathbf {s}\,, \end{aligned}$$

where *a* is the sphere’s radius and $q_{\,\ell m}$ are regular multipole moments of the charge distribution $\rho (\mathbf {s})$.

As is expected for a spherical harmonics expansion for a Helmholtz equation, the radial coefficients are the modified spherical Bessel functions: $i_\ell (z)$ are the modified spherical Bessel functions of the first kind with $i_\ell (z) = (i)^{-\ell } j_\ell (i\,z)$, while $k_\ell (z)$ are the modified spherical Bessel functions of the second kind with $k_\ell (z) = -(i)^{\ell } h^\mathrm{(1)}_\ell (i\,z)$. The functions are calculated at the dimensionless radius of one of the spheres $\kappa a_1$ or $\kappa a_2$.

The functions $\mathcal {K}(\ell , x)$ and $\mathcal {I}(\ell , x)$ are introduced solely for the brevity of notation; they depend on the dielectric constant of the medium $\epsilon _o$ and on the dielectric constant $\epsilon$ of the corresponding sphere:

7 $$\begin{aligned} \mathcal {K}(\ell , x)= & {} x \left[ \ell \left( \epsilon -\epsilon _o\right) + \frac{x\, k_{\ell +1}(x)}{ k_\ell (x)} \epsilon _o \right] ,\nonumber \\ \mathcal {I}(\ell , x)= & {} x \left[ \ell \left( \epsilon -\epsilon _o\right) - \frac{x \, i_{\ell +1}(x)}{ i_\ell (x)} \epsilon _o \right] . \end{aligned}$$

Finally, the function $\mathcal {H}_{\ell m \ell _1} (\kappa L )$ is defined as:

8 $$\begin{aligned} \mathcal {H}_{\ell m \ell _1} (\kappa L)= & {} (-1)^m \sqrt{(2\ell +1)\left( 2\ell _1+1\right) }\nonumber \\&\sum _{\ell _2=|\ell -\ell _1|}^{\ell +\ell _1} C^{\ell _2 \,0}_{\ell \, 0 \,\ell _1 0}\, C^{\ell _2 \,0}_{\ell \, m\, \ell _1\, -m}\, k_{\ell _2}(\kappa L),\nonumber \\ \end{aligned}$$

it is a reduced version of a more general factor $\mathcal {H}_{\ell m \ell _1 m_1} (\kappa \mathbf {L}_{k\rightarrow j} )$, Eq. (36), that in the general formalism takes care of the mutual location and orientation of different spheres. Note, the quantity $\mathcal {H}_{\ell m \ell _1} (\kappa L )$ is symmetric with respect to the interchange $\ell \leftrightarrow \ell _1$, allowing for a more efficient numerical implementation and further simplifications.

With the above definitions, the system of linear equations (5) with an appropriately chosen maximum number of components $\ell _\mathrm{max}$ can be solved, and the unknown components of the scaled net charge distributions $\breve{Q}_{\ell m}^{1+}$ and $\breve{Q}_{\ell m}^{2+}$ can thus be determined.

Now, with the known $\breve{Q}_{\ell m}^{1+}$ and $\breve{Q}_{\ell m}^{2+}$, the interaction energy can be found as:

9 $$\begin{aligned} U_\mathrm{int}= & {} \frac{\kappa }{2} \sum _{\ell m} \left[ i_\ell \left( \kappa a_1\right) \, k_\ell \left( \kappa a_1\right) \, \overline{Q}^{1*}_{\ell m}\, \breve{Q}^{1+}_{\ell m} - \frac{\overline{Q}^{1*}_{\ell m} \, \overline{Q}^1_{\ell m} }{ \mathcal {K}\left( \ell , \kappa a_1\right) } \right] \nonumber \\&+\frac{\kappa }{2} \sum _{\ell m} \left[ i_\ell \left( \kappa a_2\right) \, k_\ell \left( \kappa a_2\right) \, \overline{Q}^{2*}_{\ell m}\, \breve{Q}^{2+}_{\ell m} - \frac{\overline{Q}^{2*}_{\ell m} \, \overline{Q}^2_{\ell m} }{ \mathcal {K}(\ell , \kappa a_2)} \right] \nonumber \\&+ \frac{\kappa }{2} \sum _{\ell m} \sum _{\ell _1}\, (-1)^{\ell _1}\, \mathcal {H}_{\ell m \ell _1} (\kappa L) \,i_l\left( \kappa a_1\right) \, i_{\ell _1} \left( \kappa a_2\right) \nonumber \\&\quad \left[ \overline{Q}^{1*}_{\ell m} \, \breve{Q}^{2+}_{\ell _1 m} + \breve{Q}^{1+}_{\ell m}\, \overline{Q}^{2*}_{\ell _1 m} \right] . \end{aligned}$$

Note that Eqs. (5) and (9) present a general, rigorous at the Debye–Hückel level, formalism for determining the induced charges and interaction energies for the case of two dielectric spheres in an ionic solution. There are no restrictions on the free charge distributions $\overline{Q}^{1}_{\ell m}$ and $\overline{Q}^{2}_{\ell m}$.

The components of the net charge distributions $\breve{Q}_{\ell m}^{1+}$ and $\breve{Q}_{\ell m}^{2+}$ can also be used to determine the electrostatic potential between the spheres as:

10 $$\begin{aligned} \Phi \left( \mathbf {r}_1\right)= & {} \sqrt{4\pi } \, \kappa \, \sum _{\ell m} \left[ k_\ell (\kappa r_1)\, Y_{\ell m} (\hat{r}_1) \,\, i_\ell (\kappa a_1)\, \breve{Q}^{1+}_{\ell m}\right. \nonumber \\&+ \left. i_\ell \left( \kappa r_1\right) \, Y_{\ell m} \left( \hat{r}_1\right) \sum _{\ell _1} (-1)^{\ell _1}\, i_{\ell _1}\left( \kappa a_2\right) \right. \nonumber \\&\left. \quad \mathcal {H}_{\ell m \ell _1} (\kappa L) \; \breve{Q}^{2+}_{\ell _1 m} \right] . \end{aligned}$$

Here $\mathbf {r}_1$ is the point where the potential is to be found. We chose the system of coordinates to be associated with the first sphere, but the second sphere’s coordinates can also be used with the appropriate change of indices. The direction of the vector $\mathbf {r}_1$ can be arbitrary, but the distance where Eq. (10) is valid is limited by the inequality $a_1< r_1 < L- a_2$.

**Fig. 2:**
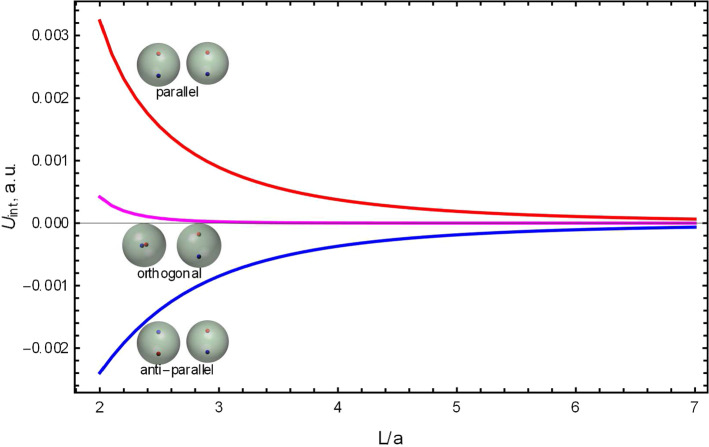
The interaction energies in atomic units between two spheres with off-center point charges. Each sphere has two equal in magnitude and opposite in sign point charges (the net charge is zero), shifted from the center in opposite directions by half of the radius. The dipoles are orthogonal to the line connecting the centers of the spheres. The interaction energy is calculated for three different orientations of these dipoles: parallel (red), anti-parallel (blue) and perpendicular (magenta). Half the sum of red and blue curves (the asymmetry of repulsion and attraction) also yield the magenta line exactly. The dimensionless screening strength is $\kappa a = 0.1$. The radii and point charges are one in atomic units, the dielectric constants inside the spheres are $\epsilon _1 = \epsilon _2 = 4$, and $\epsilon _o = 80$ for the medium

As an illustration of capabilities of this general method, let us consider electrostatic interaction in a non-axially symmetric system. In Fig. 2, we plot interaction energies between two spheres with physical dipoles oriented perpendicular to $\mathbf {L}_{1\rightarrow 2}$. The dipoles are created by two point charges, shifted from the corresponding sphere’s center by half of its radius. The charges are equal in magnitude and opposite in sign, so the net charge of each sphere is zero. The interaction energy is calculated for three different orientations of these dipoles: parallel, anti-parallel and orthogonal. As expected, the spheres with parallel dipoles repel and the spheres with anti-parallel dipoles attract.

There are two noteworthy features, however, in Fig. 2. The first one is the asymmetry in repulsion of parallel dipoles and attraction of anti-parallel ones. This stems from asymmetric dielectric screening [52], a known effect in the interaction between dielectric objects whereby either repulsion (if $\epsilon _\mathrm{in} < \epsilon _\mathrm{out}$) or attraction (if $\epsilon _\mathrm{in} > \epsilon _\mathrm{out}$) is enhanced as compared to interaction of point charges. For a discussion and an application of this effect to interaction of dielectric spheres, see, e.g., Refs. [50, 57]. The second noteworthy feature is the fact that the interaction between two orthogonal dipoles (shown in magenta) is not zero, as one might have anticipated. Indeed, if the dielectric spheres were absent, then the interaction would vanish. However, the asymmetric dielectric screening is an independent feature due to the dielectric mismatch between the spheres and the solvent and persists even when the interactions of the point charges cancel.

### Two spheres with axially symmetric charge distributions

When axial symmetry is lacking, such as in the example of two dipoles considered above, all the components of the scaled free and net charge distributions, $\overline{Q}_{\ell m}$ and $\breve{Q}^{+}_{\ell m}$, can be nonzero. This means that one has to solve the complete system of $2(\ell _\mathrm{max}+1)^2$ linear equations (5). However, in the case of axial symmetry, only the $m=0$ components $\overline{Q}_{\ell 0}$ and $\breve{Q}^{+}_{\ell 0}$ will be nonzero. The new system will only have $2(\ell _\mathrm{max}+1)$ equations, and the calculations will simplify drastically.

System (5) is then reduced to:

11 $$\begin{aligned} \overline{Q}^1_{\ell 0}= & {} i_\ell \left( \kappa a_1\right) \, k_\ell \left( \kappa a_1\right) \, \mathcal {K}\left( \ell , \kappa a_1\right) \, \breve{Q}^{1+}_{\ell \,0} \nonumber \\&+ \sum _{\ell _1} (-1)^{\ell _1}\, i_\ell \left( \kappa a_1\right) \, i_{\ell _1}\left( \kappa a_2\right) \, \mathcal {I}\left( \ell , \kappa a_1\right) \, \mathcal {H}_{\ell \, 0\, \ell _1} (\kappa L) \; \breve{Q}^{2+}_{\ell _1 0} \nonumber \\ \overline{Q}^2_{\ell 0}= & {} i_\ell \left( \kappa a_2\right) \, k_\ell \left( \kappa a_2\right) \, \mathcal {K}\left( \ell , \kappa a_2\right) \, \breve{Q}^{2+}_{\ell \, 0} \nonumber \\&+ \sum _{\ell _1} (-1)^{\ell }\, i_\ell \left( \kappa a_2\right) \, i_{\ell _1}\left( \kappa a_1\right) \, \mathcal {I}\left( \ell , \kappa a_2\right) \, \mathcal {H}_{\ell \, 0\, \ell _1} (\kappa L)\, \breve{Q}^{1+}_{\ell _1 0}. \nonumber \\ \end{aligned}$$

For further simplification, one can use explicit expressions for Clebsch–Gordan coefficients with zero momentum projections [58] to obtain $\mathcal {H}_{\ell \,0\,\ell _1}(\kappa L)$ in terms of factorials,

12 $$\begin{aligned} \mathcal {H}_{\ell \,0\,\ell _1}(\kappa L)&\!\!=&\!\! \sqrt{(2\ell +1)(2\ell _1+1)}\, \nonumber \\&\!\!\!\times \sum _{\ell _2=|\ell -\ell _1|}^{\ell +\ell _1} \!\!\! \left( \frac{\sqrt{2\ell _2+1}\, g!\, \sqrt{(2g-2\ell )!\,(2g-2\ell _1)!\,(2g-2\ell _2)!}}{(g-\ell )!\,(g-\ell _1)!\,(g-\ell _2)! \sqrt{(2g+1)!}} \right) ^2 k_{\ell _2}(\kappa L), \end{aligned}$$

where $2g =\ell +\ell _1+\ell _2$ must be even.

Moreover, it is now easy to find the force acting on each sphere, because for charge distributions axially symmetric around the vector $\mathbf {L}$, the force is directed along $\mathbf {L}$. In this case, in order to determine the force, we can take a derivative of the interaction energy with respect to the distance *L* (instead of taking a gradient as in the most general case):

13 $$\begin{aligned} F\! =\! -\frac{\partial U_\mathrm{int}}{\partial L}\!=\! & {} \! \frac{\kappa ^2}{2} \sum _{\ell } \left[ i_\ell (\kappa a_1)\, k_\ell (\kappa a_1)\, \overline{Q}^{1*}_{\ell 0}\, \left( -\frac{\partial \breve{Q}^{1+}_{\ell 0}}{\partial (\kappa L)}\right) \right. \nonumber \\&\left. + i_\ell (\kappa a_2)\, k_\ell \left( \kappa a_2\right) \, \overline{Q}^{2*}_{\ell 0}\, \left( -\frac{\partial \breve{Q}^{2+}_{\ell 0}}{\partial (\kappa L)}\right) \right] \nonumber \\&\!+ \frac{\kappa ^2}{2} \sum _{\ell } \sum _{\ell _1}\, (-1)^{\ell _1}\, \left( -\frac{\mathrm {d} \mathcal {H}_{\ell \, 0\, \ell _1} (\kappa L)}{\mathrm {d} (\kappa L)}\right) \nonumber \\&\,i_l(\kappa a_1)\, i_{\ell _1} (\kappa a_2)\, \left[ \overline{Q}^{1*}_{\ell 0} \, \breve{Q}^{2+}_{\ell _1 0} + \breve{Q}^{1+}_{\ell 0}\, \overline{Q}^{2*}_{\ell _1 0}\right] \nonumber \\&\!+ \frac{\kappa ^2}{2} \sum _{\ell } \sum _{\ell _1}\, (-1)^{\ell _1}\, \mathcal {H}_{\ell \, 0\, \ell _1} (\kappa L) \, i_l(\kappa a_1)\, i_{\ell _1} (\kappa a_2) \nonumber \\&\! \times \left[ \overline{Q}^{1*}_{\ell 0} \left( -\frac{\partial \breve{Q}^{2+}_{\ell _1 0}}{\partial (\kappa L)}\right) +\left( -\frac{\partial \breve{Q}^{1+}_{\ell 0}}{\partial (\kappa L)}\right) \, \overline{Q}^{2*}_{\ell _1 0} \right] . \nonumber \\ \end{aligned}$$

For computational purposes, one can again express the derivative of the modified spherical Bessel as in Eq. (41):

14 $$\begin{aligned} \frac{\partial k_\ell (x)}{\partial x} = \frac{\ell \, k_{\ell }(x) - x \, k_{\ell +1}(x)}{x}. \end{aligned}$$

The derivatives of the components of the scaled net charge distributions $\breve{Q}^+_{\ell \,0}$ can be found by taking derivatives of both parts of Eq. (11):

15 $$\begin{aligned} 0= & {} i_\ell (\kappa a_1)\, k_\ell (\kappa a_1)\, \mathcal {K}(\ell , \kappa a_1) \left( \frac{\partial \breve{Q}^{1+}_{\ell \, 0}}{\partial (\kappa L)}\right) \nonumber \\&+ \sum _{\ell _1} (-1)^{\ell _1}\, i_\ell (\kappa a_1) \, i_{\ell _1}(\kappa a_2)\, \mathcal {I}\left( \ell , \kappa a_1\right) \nonumber \\&\left[ \frac{\mathrm {d} \mathcal {H}_{\ell \, 0\, \ell _1} (\kappa L)}{\mathrm {d} (\kappa L)}\, \breve{Q}^{2+}_{\ell _1\, 0} + \mathcal {H}_{\ell \, 0\, \ell _1} (\kappa L) \left( \frac{\partial \breve{Q}^{2+}_{\ell _1 0}}{\partial (\kappa L)}\right) \right] \nonumber \\ 0= & {} i_\ell (\kappa a_2)\, k_\ell (\kappa a_2)\, \mathcal {K}(\ell , \kappa a_2) \left( \frac{\partial \breve{Q}^{2+}_{\ell \,0}}{\partial (\kappa L)}\right) \nonumber \\&+ \sum _{\ell _1} (-1)^{\ell }\, i_\ell (\kappa a_2) \, i_{\ell _1}(\kappa a_1)\, \mathcal {I}\left( \ell , \kappa a_2\right) \nonumber \\&\left[ \frac{\mathrm {d} \mathcal {H}_{\ell \, 0\, \ell _1} (\kappa L)}{\mathrm {d} (\kappa L)}\, \breve{Q}^{1+}_{\ell _1\,0} + \mathcal {H}_{\ell \, 0\, \ell _1} (\kappa L) \left( \frac{\partial \breve{Q}^{1+}_{\ell _1\,0}}{\partial (\kappa L)}\right) \right] \nonumber \\ \end{aligned}$$

Assuming that the system of linear equations (11) has already been solved and introducing quantities

16 $$\begin{aligned} \Theta ^1_{\ell }\equiv & {} (-1)^{\ell }\, i_\ell \left( \kappa a_2\right) \, \mathcal {I}\left( \ell , \kappa a_2\right) \sum _{\ell _1} i_{\ell _1}\left( \kappa a_1\right) \, \mathcal {H}'_{\ell \,0\,\ell _1} (\kappa L)\, \breve{Q}^{1+}_{\ell _10}\nonumber \\ \Theta ^2_{\ell }\equiv & {} i_\ell \left( \kappa a_1\right) \, \mathcal {I}\left( \ell , \kappa a_1\right) \sum _{\ell _1} (-1)^{\ell _1}\, i_{\ell _1}\left( \kappa a_2\right) \, \mathcal {H}'_{\ell \,0\,\ell _1} (\kappa L)\, \breve{Q}^{2+}_{\ell _1 0}, \nonumber \\ \end{aligned}$$

we obtain a system of linear equations for the negative derivatives:

17 $$\begin{aligned} \Theta ^2_{\ell }= & {} i_\ell \left( \kappa a_1\right) \, k_\ell \left( \kappa a_1\right) \, \mathcal {K}\left( \ell , \kappa a_1\right) \, \left( -\frac{\partial \breve{Q}^{1+}_{\ell \, 0}}{\partial (\kappa L)}\right) \nonumber \\&+ \sum _{\ell _1} (-1)^{\ell _1}\, i_\ell \left( \kappa a_1\right) \, i_{\ell _1}\left( \kappa a_2\right) \, \mathcal {I}\left( \ell , \kappa a_1\right) \, \mathcal {H}_{\ell \,0\,\ell _1} (\kappa L)\nonumber \\&\left( -\frac{\partial \breve{Q}^{2+}_{\ell _1 0}}{\partial (\kappa L)}\right) \nonumber \\ \Theta ^1_{\ell }= & {} i_\ell \left( \kappa a_2\right) \, k_\ell \left( \kappa a_2\right) \, \mathcal {K}\left( \ell , \kappa a_2\right) \, \left( -\frac{\partial \breve{Q}^{2+}_{\ell \, 0}}{\partial (\kappa L)}\right) \nonumber \\&+ \sum _{\ell _1} (-1)^{\ell }\, i_\ell \left( \kappa a_2\right) \, i_{\ell _1}(\kappa a_1)\, \mathcal {I}\left( \ell , \kappa a_2\right) \, \mathcal {H}_{\ell \,0\,\ell _1} (\kappa L)\nonumber \\&\left( -\frac{\partial \breve{Q}^{1+}_{\ell _1 0}}{\partial (\kappa L)}\right) \end{aligned}$$

Note that systems (11) and (17) have the same matrix of coefficients; only the source terms are different. This makes a computational implementation of force calculation much more efficient.

This increased computational efficiency, appearing in the case of axially symmetric systems due to the reduced size of matrix (11) and the possibility of its re-purposing for determining the force, allows calculating electrostatic interactions in a rigorous fashion down to very short distances, almost to the touching point, where the number of contributing terms may increase to hundreds.

Janus particles (see, e.g., a recent paper [59]) could serve as a practical example of such an axially symmetric system. Within the present formalism, they are easily modeled as two patches of positive and negative charge, uniformly distributed over a spherical cap of a polar angle $\theta _0$. In this case, the spherical components of the free charge are given by:

18 $$\begin{aligned} \overline{Q}_{00}= & {} 0\nonumber \\ \overline{Q}_{\ell m}= & {} \left[ 1-(-1)^\ell \right] \frac{q}{\sqrt{2\ell +1}}\nonumber \\&\, \frac{P_{\ell -1}\left( \cos \theta _0\right) - P_{\ell +1}\left( \cos \theta _0\right) }{1-\cos \theta _0} \, \delta _{m\, 0}, \quad \ell >0,\nonumber \\ \end{aligned}$$

where $P_\ell (x)$ are Legendre polynomials. It is interesting to note that for reasonable values of the opening polar angle $\theta _0$ the scaled free charge components are close to those of a simple dipole oriented along the *z*-axis, as can be seen from the small-$\theta _0$ expansion

19 $$\begin{aligned} \overline{Q}_{\ell m} = \left[ 1-(-1)^\ell \right] \sqrt{2\ell +1} \, q\, \left[ 1-\frac{\ell (\ell +1)}{4}(1-\cos \theta _0) \right] \, \delta _{m\, 0}. \end{aligned}$$

Our numerical results confirm this conclusion.

Another instance of a directly observable situation where an axially symmetric charge distribution might prove useful is modeling interaction of two dielectric beads that are attached to a support. For example, in an optical tweezers experiment one of the colloid particles is held in place by a pipette [14], which makes an otherwise spherically uniform charge distribution only axially symmetric. (Our calculations show that the presence of a pipette in these kinds of experiment would be important for low $\kappa a$ regimes.)

### Two spheres with point charges at their centers

Finally, let us write down the expressions for the interaction energy and the force for the important case when both spheres have only one point charge located at their center. Let’s denote these charges $q_1$ and $q_2$. Then, according to Eq. (6),

20 $$\begin{aligned} \overline{Q}^k_{\ell \, m}= q_k \, \delta _{\ell \, 0}\, \delta _{m 0}. \end{aligned}$$

The interaction energy now has two simple terms centered at each sphere and only one summation in the cross term. Indeed, only the $\ell =0$, $m=0$ components in the first two sums in Eq. (9) survive, while the compounded sum over $\ell$, *m* and $\ell _1$ in the third term turns into two independent sums over $\ell$ and $\ell _1$ (which can be combined by renaming $\ell _1$ back to $\ell$).

Using the explicit expressions for zero-order modified spherical Bessel functions, $k_0(\kappa a) = e^{- \kappa a}/(\kappa a)$, $i_0(\kappa a)= \sinh (\kappa a)/(\kappa a)$, and noting that $\mathcal {H}_{\ell \, 0\, 0} (\kappa L) = \sqrt{(2\ell +1)}\, k_{\ell }(\kappa L)$, we obtain

21 $$\begin{aligned} U_\mathrm{int}\left( q_1;q_2\right)= & {} \frac{q_1}{2a_1} \left[ \frac{\sinh \left( \kappa a_1\right) }{\kappa a_1}e^{-\kappa a_1} \breve{Q}^{1+}_{0 0} - \frac{1}{\epsilon _o} \frac{q_1 }{ 1+ \kappa a_1} \right] \nonumber \\&+\frac{q_2}{2a_2} \left[ \frac{\sinh \left( \kappa a_2\right) }{\kappa a_2}e^{-\kappa a_2} \breve{Q}^{2+}_{0 0} - \frac{1}{\epsilon _o} \frac{q_2 }{ 1+ \kappa a_2} \right] \nonumber \\&+ \frac{1}{2} \sum _{\ell } \sqrt{(2\ell + 1)} k_\ell (\kappa L) \left[ i_{\ell } \left( \kappa a_1\right) \sinh \left( \kappa a_2\right) \frac{q_2}{a_2}\, \breve{Q}^{1+}_{\ell \,0} \right. \nonumber \\&\left. + (-1)^{\ell }\, i_{\ell } (\kappa a_2)\sinh (\kappa a_1) \frac{q_1}{a_1} \, \breve{Q}^{2+}_{\ell \,0} \right] . \end{aligned}$$

Consequently, the force is

22 $$\begin{aligned} F= & {} -\frac{\partial U_\mathrm{int}}{\partial L} = \frac{q_1}{2a_1^2} \sinh (\kappa a_1)\, e^{-\kappa a_1} \left( -\frac{\partial \breve{Q}^{1+}_{00}}{\partial (\kappa L)}\right) \nonumber \\&+\frac{q_2}{2a_2^2} \sinh (\kappa a_2)\, e^{-\kappa a_2} \left( -\frac{\partial \breve{Q}^{2+}_{00}}{\partial (\kappa L)}\right) \nonumber \\&+ \frac{\kappa }{2} \sum _{\ell } \sqrt{(2\ell +1)}\, k'_\ell (\kappa L) \left[ i_{\ell } (\kappa a_1)\sinh (\kappa a_2) \frac{q_2}{a_2}\, \breve{Q}^{1+}_{\ell \,0}\right. \nonumber \\&\left. + (-1)^{\ell }\, i_{\ell } (\kappa a_2)\sinh (\kappa a_1) \frac{q_1}{a_1} \, \breve{Q}^{2+}_{\ell \,0}\right] \nonumber \\&+ \frac{\kappa }{2} \sum _{\ell } \sqrt{(2\ell +1)}\, k_\ell (\kappa L) \left[ i_{\ell } (\kappa a_1)\sinh (\kappa a_2) \frac{q_2}{a_2}\, \left( -\frac{\partial \breve{Q}^{1+}_{\ell \,0}}{\partial (\kappa L)}\right) \right. \nonumber \\&\left. + (-1)^{\ell }\, i_{\ell } (\kappa a_2)\sinh (\kappa a_1) \frac{q_1}{a_1} \, \left( -\frac{\partial \breve{Q}^{2+}_{\ell \,0}}{\partial (\kappa L)}\right) \right] , \end{aligned}$$

where the components of the scaled net charge distributions $\breve{Q}^+_{\ell \,0}$ are found by solving the system of equations (11) with $\overline{Q}^k_{\ell \,0}= q_k \, \delta _{\ell \, 0}$. Their derivatives are found by solving system (17).

## Comparison with the DLVO theory

The presented formalism is exact and therefore allows a quantitative characterization of errors arising from using the DLVO approximation. Within the DLVO approximation, the electrostatic interaction energy is given by the term:

23 $$\begin{aligned} U_\mathrm{int}^\mathrm{DLVO} = \frac{q_1 \, q_2}{\epsilon _o L} \frac{e^{-\kappa D}}{(1+\kappa a_1)(1+\kappa a_2)}, \end{aligned}$$

where $D=L-a_1-a_2$ is the separation between the spheres’ surfaces. Generally, the accuracy that can be achieved with the DLVO theory is governed by the number of terms in the polynomial expansion of the surface charge that contribute significantly to the interaction energy. For large separations and/or very short screening lengths, the $\ell =0$ term dominates and the DLVO approximation is accurate. For short separations and moderate screening lengths, however, the DLVO approximation can lead to large errors.

**Fig. 3:**
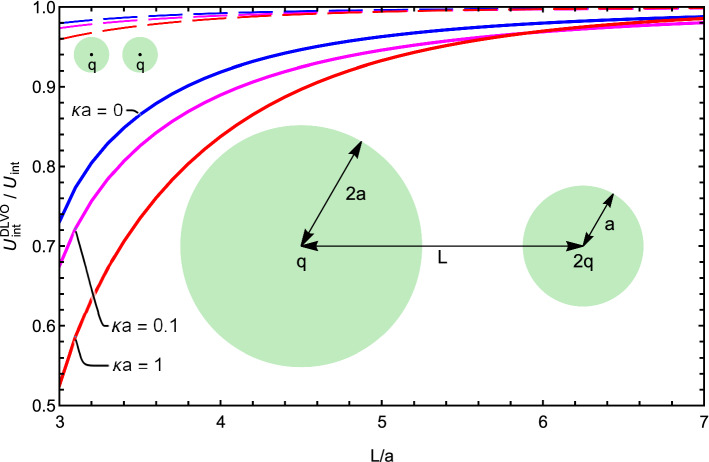
The ratio of the approximate DLVO interaction energy to the fully converged $U_\mathrm{int}$ of Eq. (21) as a function of distance between the centers of the spheres *L* for several values of the dimensionless screening strength $\kappa a$: $\kappa a = 0$ (blue lines), $\kappa a = 0.1$ (magenta lines), $\kappa a = 1$ (red lines). The solid lines correspond to the asymmetric geometry where the first sphere has a charge *q* and a radius 2*a*, while the second sphere has the double charge 2*q* and a smaller radius *a*. The dashed lines correspond to the fully symmetric situation—both spheres have charge *q* and radius *a*. The charge *q* and the radius *a* are one in atomic units; the dielectric constants inside the spheres are $\epsilon _1 = \epsilon _2 = 4$, and $\epsilon _o = 80$ for the medium

**Fig. 4:**
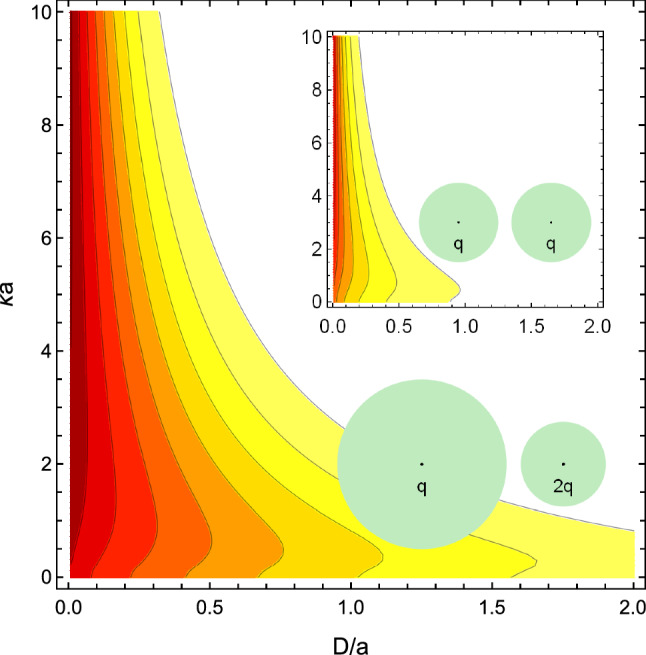
The ratio of the approximate DLVO interaction force to the fully converged force of Eq. (22) in the plane of surface-to-surface separation *D* and screening strength $\kappa a$ for the two identical spheres (inset) and for two spheres with different radii and charges. The parameters of the system are the same as in Fig. 3. The contours represent (going from the top right corner toward the bottom left corner) the ratios of 0.9, 0.8, 0.7, 0.6, 0.5, 0.4, 0.3, and 0.2

In Fig. 3, we plot the ratio of $U_\mathrm{int}^\mathrm{DLVO}$ to the full interaction energy $U_\mathrm{int}$ given by Eq. (21) for three dimensionless screening strengths $\kappa a = 0, 0.1, 1$. As the strength of screening increases, the DLVO approximation becomes worse at small separations of the spheres, while getting better at large separations. The accuracy of the DLVO theory also suffers for asymmetric charge distribution and asymmetric radii (compare the solid and the dashed lines in the figure). Indeed, for two identical spheres only the first two terms in the expansion are enough to achieve accuracy of one per cent even at the point where the spheres touch, the configuration that presents the most stringent test of accuracy and convergence. However, for highly asymmetric radii and charge distributions one may need a hundred terms to get to the relative error of $10^{-3}$.

While the inaccuracies of the DLVO theory in interaction energies are usually moderate, they are much higher for the interaction forces, the physical quantities that are directly measured by atomic force microscopy or optical tweezers experiment, see, e.g., Refs. [14, 60]. As an illustration, in Fig. 4 we plot the ratio of the DLVO force to the exact, fully converged interaction force of Eq. (22), for the two systems for which the ratio of energies is plotted in Fig. 3. For a more comprehensive perspective, we plot the force ratio in the plane of separations *D* and screening strengths $\kappa a$. The region of the parameter space for which the ratio of the DLVO force to the exact force is greater than 0.9 (DLVO accurate within roughly 10% error) has a white background. The dark red background corresponds to fivefold errors of the DLVO approximation (the 0.2 ratio). For large separations *D* and values of $\kappa a$ (the top right corner), the ratio of the DLVO’s result to the exact one is nearly one, while the ratio decreases (for the asymmetric system—dramatically), toward the area of small separations and weak screening.

**Fig. 5:**
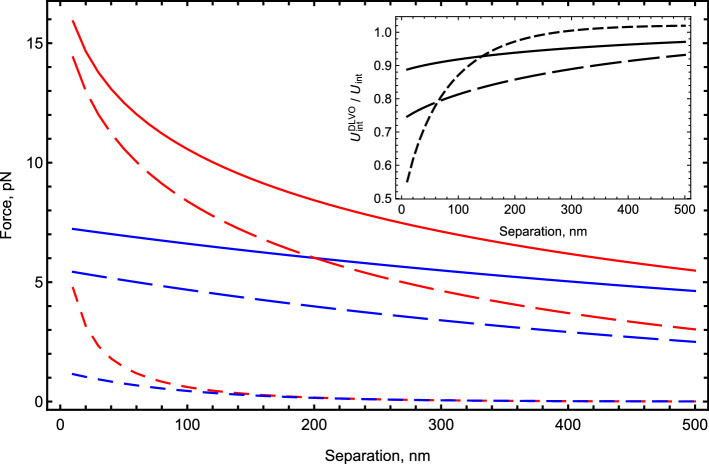
The interaction force between two colloidal polystyrene particles ($\epsilon _1 = \epsilon _2 = 2.6$) of radius $a_1=a_2=1 \,\upmu$m uniformly charged on their surfaces to $q_1=q_2= 10^5$ elementary charges in ionic solutions ($\epsilon _o = 78.3$) of three different screening strengths: $\kappa a = 0.1$ (solid lines), $\kappa a = 1$ (long dashed lines), $\kappa a = 10$ (short dashed lines). The forces are calculated using the DLVO approximation (blue lines) and the full formalism (22) (red lines). Accounting for the interactions with $\ell > 0$ increases the repulsive forces several fold. In the inset, we plot the ratio of the interaction energies for this case

We also present interaction forces for conditions similar to those of experiments measuring forces between two colloidal particles [14], as shown in Fig. 5. For a 1 $\upmu$m particle charged to $10^5$ elementary charges (0.008 elementary charges per nm squared), at small separations the forces again differ by several fold, while the interaction energies differ by only several tens of per cent.

We conclude, therefore, that the DLVO approximation provides accurate interaction energies and forces for large separations and for strong screening, while for short separation, weak screening and for asymmetric charge/radii combinations the DLVO theory predictions can be misleading. This is also true for systems involving multiple interacting spheres, where many-body effects, considered in the next section, can play an important role.

## Many-body effects

Obtaining a general solution to the problem of multiple dielectric spheres in an electrolyte, not restricted by any symmetry considerations, is a much harder problem, as compared to calculating the interaction between just two spheres. Not only does the general solution require more computational resources, its results are more difficult to predict and analyze. That is why it is so tempting to approximate the interaction within a set of spheres by the sum of their pairwise interactions. The difference between the results obtained within the pairwise approximation and within a full formalism is frequently referred to as “many-body effects”. In any particular physical model in which many-body effects are possible, the role they play might range from vital to, on the contrary, a negligible one. In this section, we use the general formalism [39] to study the importance of many-body interactions for systems of three and four dielectric spheres in an electrolyte.

Earlier it was demonstrated [61] that many-body effects are important in interactions of several dielectric spheres in vacuum, and their significance increases with the number of interacting spheres. For example, if three positively charged spheres were arranged in an equilateral triangle, the charge density on each sphere had a broad distribution of negative surface charge centered in the direction of the triangle’s center. On top of that broad distribution, there were two additional smaller peaks oriented toward the other two spheres. However, within the pairwise approximation the negative surface charge distribution was less pronounced and, moreover, had a simple singly-peaked sinusoidal shape, a result of a superposition of two sinusoidal pairwise charge distributions. Thus, the pairwise approximation in this example failed both in predicting correct magnitude of the surface charge density and in predicting possible fine features of the surface charge distribution. Such inaccuracies in the surface charge density led to substantial errors in the interaction energies found within the pairwise approximation [61]. Let us now examine how the presence of mobile ions in the dielectric medium influences the significance of the many-body effects.

**Fig. 6:**
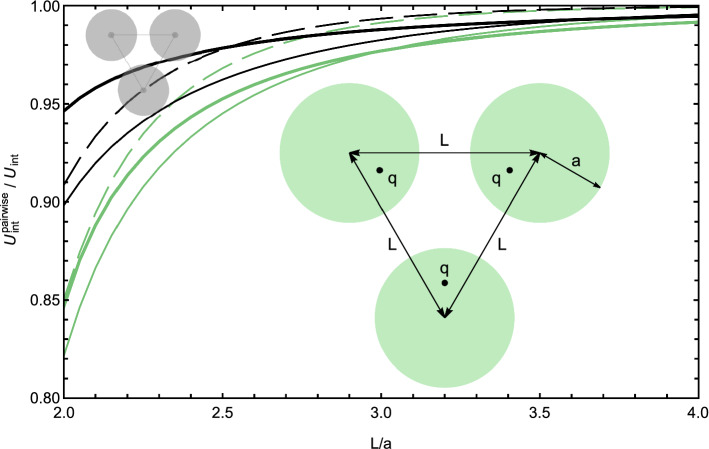
The ratio of the interaction energies between three spheres forming an equilateral triangle with the side *L*, calculated as a solution of the consistent many-body Eq. (44) and as a sum of three pairwise interactions between two spheres. The ratio is calculated for point charges at the center of each sphere (black lines marked by a gray geometry sketch) and for point charges shifted by half the radius from the center of each sphere toward the geometric center of the system (green lines and the green geometry sketch). Three dimensionless screening strengths $\kappa a = 0.1$ (thick lines), $\kappa a = 1$ (thin lines) and $\kappa a = 2$ (dashed thin lines) were used. The charge *q* and the radius *a* are one in atomic units; the dielectric constants inside the spheres are $\epsilon _1 = \epsilon _2 = 4$, and $\epsilon _o = 80$ for the medium. The cutoff values of $\ell _\mathrm{max}=20$ (shifted charges) and $\ell _\mathrm{max}=15$ (centered charges) were used to achieve convergence to the relative accuracy level of $10^{-3}$

From the physical viewpoint, it is clear that at large distances, $\kappa L \gg 1$, pairwise interactions should provide a very accurate estimate of the total interaction energy and the shorter the Debye length becomes, the more accurate this estimate gets. This is due to the fact that at large distances electrostatic interactions are dominated by the $\ell =0$ term, while the contribution of higher-order terms is small and, moreover, gets screened more and more effectively with shorter Debye length. With respect to another length scale, $\kappa a$, in the limit of a very short Debye length, $\kappa a \gg 1$, the direct Coulomb interaction term $\ell =0$ would also dominate even as the spheres touch, since the mobile ions would completely screen the presence of the other spheres, suppressing the manifestation of many-body effects. However, it is not obvious what happens in a situation when the Debye length is comparable to the radii of the spheres, $\kappa a \sim 1$, and the spheres are not too far apart, $a \sim L$.

Our calculations show that in this regime, the presence of mobile ions in the medium actually enhances the importance of collective effects, many more higher multipoles contribute significantly to the interaction energy, and the quality of the pairwise approximation gets much worse.

This phenomenon is clearly seen in Fig. 6. In this figure, we plot, for a system of three spheres, the ratio of interaction energies found within the pairwise approximation (the sum of three identical pairwise interactions between the spheres) and within the rigorous formalism, given by Eqs. (39) and (44). The spheres form an equilateral triangle. Inside each sphere, there is a positive point charge that we position either at the sphere’s center or shift by a half of the radius toward the geometric center of the triangle. The ratio is plotted for three values of the screening strength, $\kappa a = 0.1, 1, 2$.

As anticipated, at large separations and as $\kappa$ increases, the curves move closer to 1, i.e., the pairwise approximation works better. However, at shorter distances the dependence on $\kappa a$ is not trivial. Consider the geometry where the spheres touch ($L=2a$) and the charges are at the centers of the spheres. For $\kappa a \rightarrow 0$, the error of the pairwise approximation is slightly more than 4%. It increases with $\kappa a$, reaching about 10% when $\kappa a \sim 1$ and then gradually decreases again.^2^

These errors are much bigger for the case when the point charges are shifted off the spheres’ centers toward the triangle’s center. The reason is that in this case the point charges are closer to the surfaces where the spheres touch, so the surface charge distributions are more peaked and more higher-order terms become important. In contrast, if the point charges are shifted *away* from the triangle’s center, the surface charge distribution is peaked at the “back side” of each sphere, with a wide, featureless distributions on the “front side” of the spheres, where they touch. In this case, the pairwise approximation becomes more accurate (not shown in Fig. 6). This observation is in line with the conclusion made for interactions of several spheres in non-ionic solutions [61], where the direction and amplitude of peaks in the surface charge density affected the accuracy of the pairwise approximation.

**Fig. 7:**
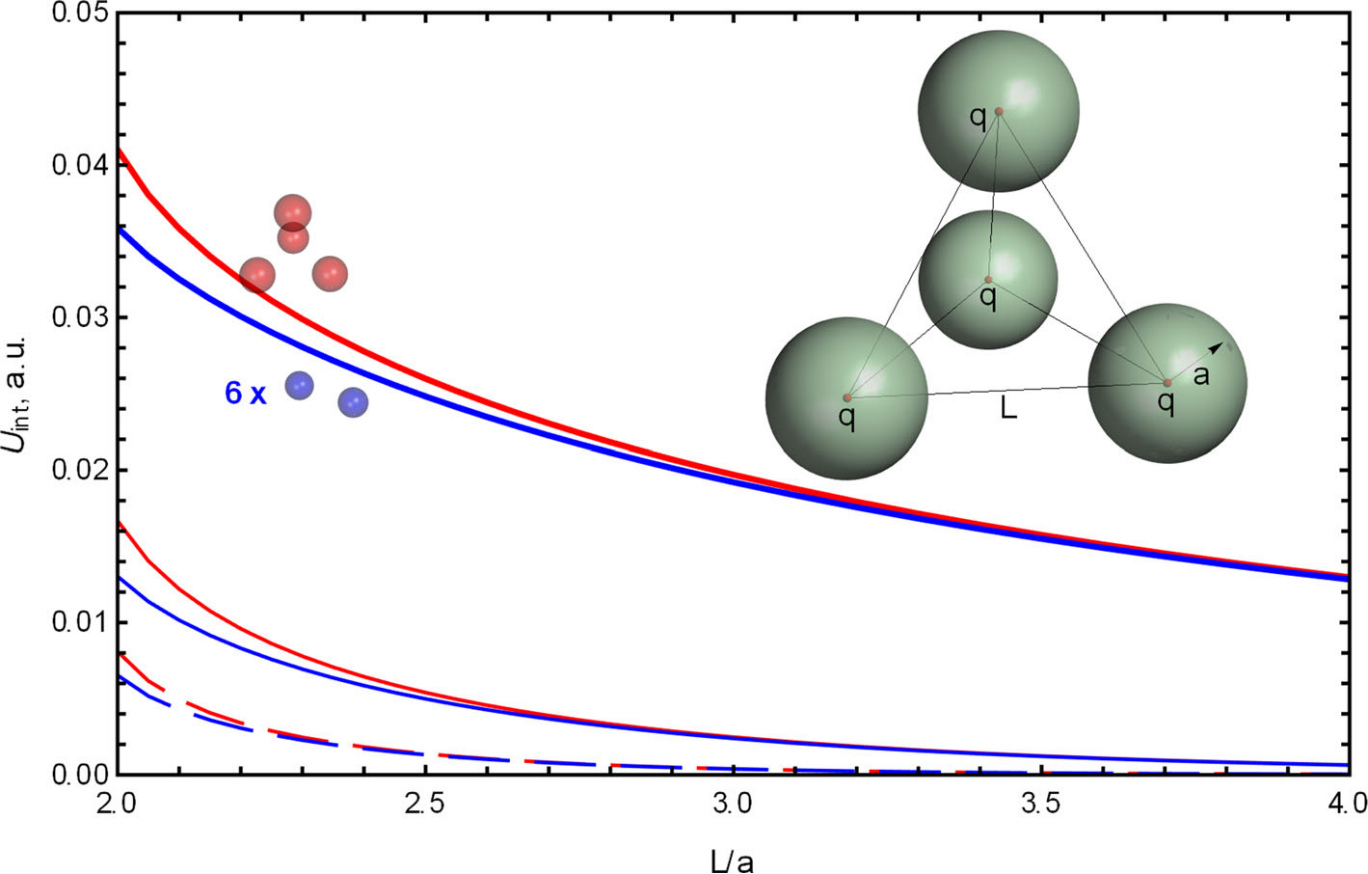
The interaction energies in atomic units between four spheres forming a tetrahedron with the side *L*. Each sphere (of a radius *a*) has a point charge *q* at its center. The interaction energies are calculated as a fully converged solution of Eq. (44) (red lines, marked by a tetrahedron icon) and as a sum of six pairwise interactions between two spheres (blue lines marked by a two-sphere icon). Three dimensionless screening strengths, $\kappa a = 0.1$ (thick lines), $\kappa a = 1$ (thin lines) and $\kappa a = 2$ (dashed thin lines), were used. The charge *q* and the radius *a* are one in atomic units, the dielectric constants inside the spheres are $\epsilon _1 = \epsilon _2 = 4$, and $\epsilon _o = 80$ for the medium

**Fig. 8:**
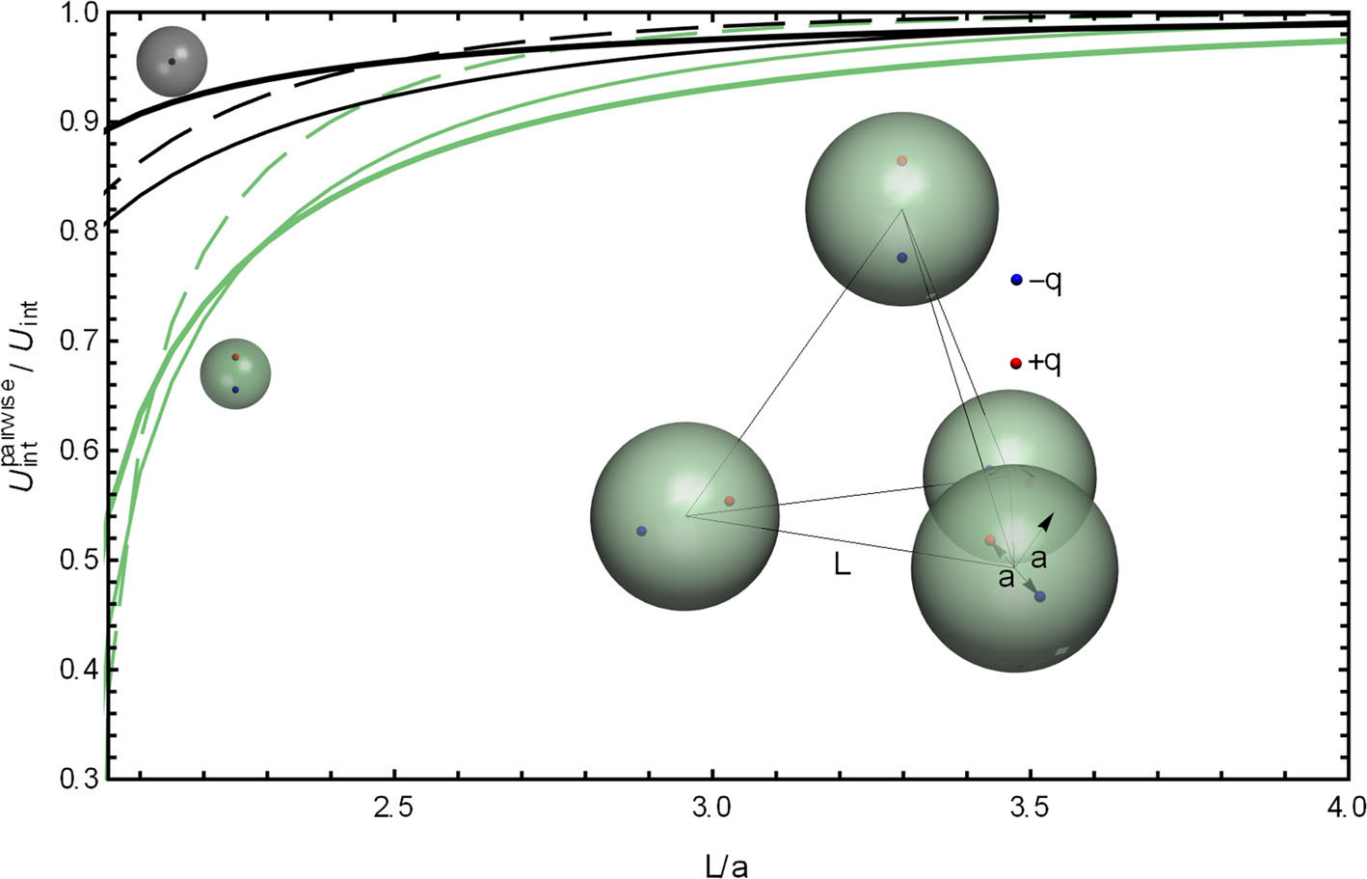
The ratio of the interaction energies between four spheres forming a tetrahedron with the side *L*, calculated as a fully converged solution of Eq. (44) and as a sum of six pairwise interactions between two spheres. The ratio is calculated for point charges at the center of each sphere (black lines marked by a gray sphere) and for physical dipoles in each sphere (green lines marked by a green sphere). The physical dipoles are formed by two point charges, equal in magnitude and opposite in sign, shifted from the center of the sphere by half radius, so the total length of each dipole is *a*, the radius of the sphere. Two of the dipoles are pointed toward the geometric center of the system by their positive end, while the other two dipoles—by their negative end. Three dimensionless screening strengths, $\kappa a = 0.1$ (thick lines), $\kappa a = 1$ (thin lines) and $\kappa a = 2$ (dashed thin lines), were used. The charge *q* and the radius *a* are one in atomic units, the dielectric constants inside the spheres are $\epsilon _1 = \epsilon _2 = 4$, and $\epsilon _o = 80$ for the medium

We observed the same behavior of the many-body effects also for a system of four spheres, see Figs. 7 and 8. The spheres are arranged in a tetrahedron, so the distances between their centers are equal. Each sphere can have either a point charge at its center or two point charges, equal in magnitude, but of opposite signs. The latter dipoles are oriented in two spheres toward the geometric center of the tetrahedron and away from it in the other two spheres.

**Fig. 9:**
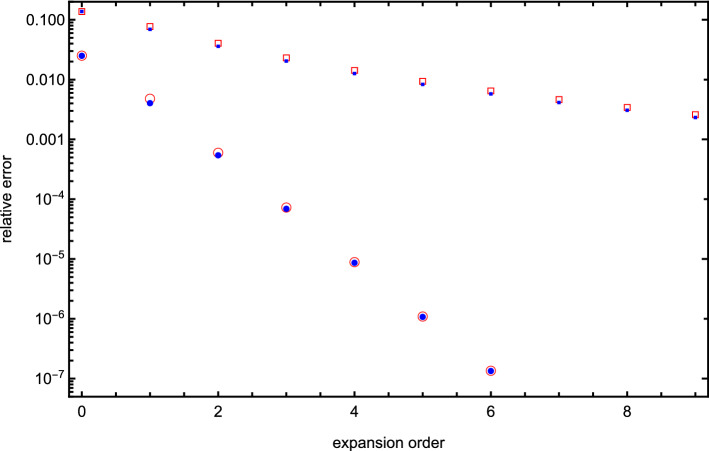
Convergence of interaction energy for two identical spheres of radii *a* and charge *q* located at the touching point $L=2a$ (squares) and with a gap of one radius between them, $L=3a$, (circles). Open squares and circles are obtained within the present formalism, while filled circles and squares are obtained using Fisher’s formalism [9]. The screening strength is $\kappa a = 0.1$, the charge *q* and the radius *a* are one in atomic units, the dielectric constants inside the spheres are $\epsilon _1 = \epsilon _2 = 4$, and $\epsilon _o = 80$ for the medium. The relative errors are calculated with respect to the fully converged value obtained for $\ell _\mathrm{max}=100$

**Fig. 10:**
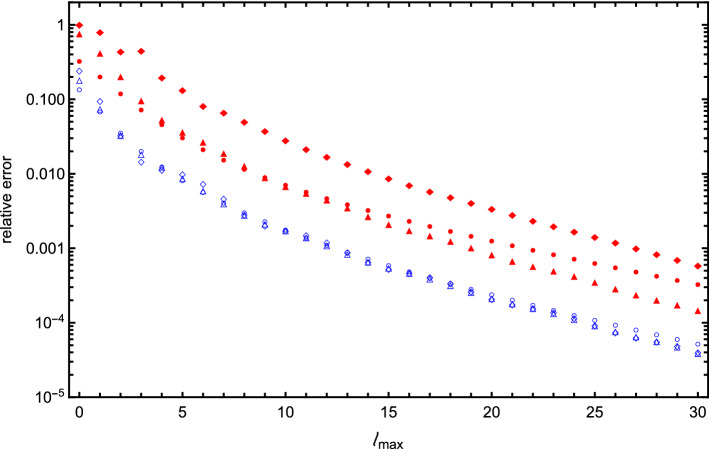
Relative error of the interaction energy as a function of the cutoff value $\ell _\mathrm{max}$ calculated at the touching point for the following systems: two identical spheres of radius *a* and point charges *q* located at their centers (blue open circle point markers), two different spheres with radii *a* and 2*a* and point charges 2*q* and *q* located at the spheres’ centers (red filled circle point markers), three identical spheres of radii *a* with point charges *q* at their centers (blue open triangle point markers), three identical spheres of radii *a* with point charges *q* shifted from their centers (red filled triangle point markers), four spheres of radii *a* with point charges *q* at their centers (blue open diamond point markers), and four spheres of radii *a* with physical dipoles arranged as in Fig. 8 (red filled diamond point markers). For all these systems, the screening strength $\kappa a = 0.1$, the charge *q* and the radius *a* are one in atomic units, the dielectric constants inside the spheres are $\epsilon _1 = \epsilon _2 = 4$, and $\epsilon _o = 80$ for the medium

**Fig. 11:**
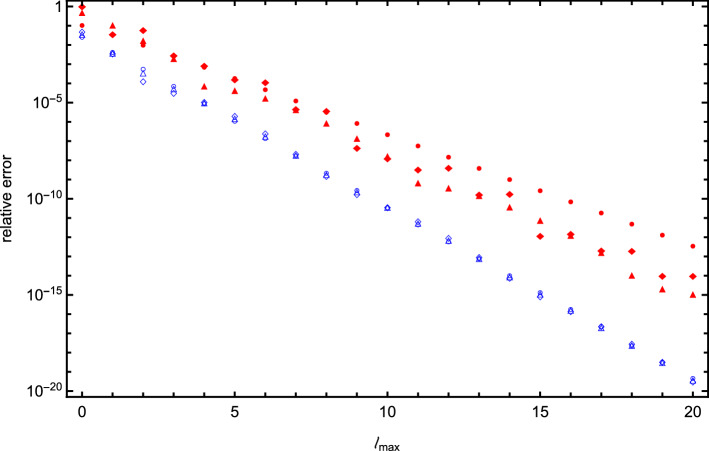
Same as in Fig. 10 but for spheres arranged with a gap of one radius *a* between their surfaces

As in the case of three spheres, for large distances the pairwise approximation is more accurate at larger $\kappa$, while at shorter distances the many-body effects are important and the pairwise approximation can greatly underestimate the interaction energy, failing completely for the case of dipoles inside the spheres.

Finally, we note that the correct account of many-body effects can only be achieved if enough terms are included in expansion of the potential/surface charge. Unfortunately, the convergence can be quite slow when spheres’ surfaces approach each other. This general characteristic of methods that rely on re-expanding spherical Bessel functions and Legendre polynomials at shifted origins is illustrated in Fig. 9. There we plot convergence of interaction energy for two identical spheres at the touching point and with a gap of one radius between the spheres. The convergence is plotted both for our method and for Fisher’s method [9], which employs a different set of basis polynomials. However, the rate of convergence is nearly identical for this system of identical spheres.

Apart from the dependence on the distance between the spheres, the convergence is generally slower for systems with large variations in the relative magnitudes of radii and charges (see, e.g., [50]) or with large variations between charge distributions within each sphere (for instance, when a point charge is located not in the center of a sphere, but close to its surface).

In Figs. 10 and 11, we illustrate this point by plotting the relative error of the interaction energy as a function of cutoff value $\ell _\mathrm{max}$ for the following pairs of systems: two identical spheres versus two spheres with different radii and charges (the system from Fig. 3), three spheres with point charges at the center versus three spheres with point charges off center (the system from Fig. 6), four spheres with point charges at the center versus four spheres with zero net charge, but with physical dipoles inside (the system from Fig. 8).

For each of these pairs, we see that variation in the charges, radii, or off-center placement of charges significantly reduces the rate of convergence. In some cases, the rate of convergence fluctuates even for large $\ell _\mathrm{max}$. For four spheres, these irregularities span only one or two expansion orders, while for three spheres we observe a more regular pattern in the fluctuation of convergence rate. We have carefully examined possible sources of numerical errors and, using arithmetic of arbitrary precision and various methods for solving the system of linear equations, verified that these peculiarities seem to be a result of a complex interplay between contributions of many different terms of the expansion, each of which has its own convergence rate and relative importance as a function of $\ell _\mathrm{max}$. Further study of the convergence patterns is needed, but care should be taken when calculating interaction energies for asymmetric systems.

## Appendix A: Violation of reciprocity in the Poisson–Boltzmann framework

To provide context, we briefly review the discussions by Onsager [3] and McQuarrie [6] of the Poisson–Boltzmann and Debye–Hückel equations. For a neutral solution of positive and negative ions in water ($\epsilon _o$), these differential equations provide $<\phi (\mathbf{r},\mathbf{r}_1)>^{(1)}$, the canonically averaged electric potential at $\mathbf{r}$ while keeping a single ion fixed at $\mathbf{r}_1$. With $\phi \equiv \sum _{i=1}^{N} q_i/(\epsilon _o |\mathbf{r}-\mathbf{r}_i|)$, one has

24 $$\begin{aligned} \nabla ^2<\phi (\mathbf{r},\mathbf{r}_1)>^{(1)}= & {} -\frac{4 \pi }{\epsilon _o}<\rho (\mathbf{r},\mathbf{r}_1)>^{(1)} \end{aligned}$$

25 $$\begin{aligned}= & {} -\frac{4 \pi }{\epsilon _o} \sum _s c_s q_s g_{1s}\left( \mathbf{r},\mathbf{r}_1\right) \end{aligned}$$

26 $$\begin{aligned}\approx & {} -\frac{4 \pi }{\epsilon _o} \sum _s c_s q_s e^{-\beta q_s <\phi (\mathbf{r},\mathbf{r}_1)>^{(1)}} \nonumber \\ \end{aligned}$$

Taking the square gradient of the expression for the canonical average of $\phi$ gives the first equality. The second equality is by the definition of the radial distribution function $g_{1s}(\mathbf{r},\mathbf{r}_1)$, where $q_s$ and $c_s$ are the charge and average concentration of the ions of species *s* and $\beta =1/k_B T$ is the inverse temperature. The final step is the *approximation* that gives the well-known Poisson–Boltzmann equation:

27 $$\begin{aligned} \nabla ^2 \phi (\mathbf{r}) = -\frac{4 \pi }{\epsilon _o} \sum _s c_s q_s e^{-\beta q_s \phi (\mathbf{r})} \end{aligned}$$

where we have without loss of generality set $\mathbf{r}_1=0$ and used streamlined notation for the potential in accordance with common practice. Expanding the exponential to first order and noting that the zeroth-order term vanishes due to charge neutrality gives the Debye–Hückel equation:

28 $$\begin{aligned} \nabla ^2 \phi (\mathbf{r}) = \kappa ^2 \phi (\mathbf{r}) \end{aligned}$$

where $\kappa ^2 = 4\pi \frac{\beta }{\epsilon _o} \sum _s c_s q_s^2$. However, we are not wedded to this expression for $\kappa$. As noted by Fisher et al. [9], the range of validity and utility of the Debye–Hückel equation may be expanded by considering $\kappa$ to be a system parameter. More recently, Janacek and Netz [10] have performed numerical studies that suggest the use of an effective screening parameter $\kappa _{\mathrm {eff}}$. The work of Alexander et al. [8] also suggests the expanded applicability of Debye–Hückel theory when using an effective $\kappa$.

Let us now consider some symmetry requirements. Generalizing the above notation so that $<\phi (\mathbf{r}_k,\mathbf{r}_j)>^{(j)}$ is the canonically averaged electric potential at a point $\mathbf{r}_k$ while keeping an ion fixed at point $\mathbf{r}_j$ (and vice versa). By imposing translational symmetry and particle permutation symmetry, the radial distribution function satisfies $g_{jk}(\mathbf{r}_j, \mathbf{r}_k) = g_{jk}(|\mathbf{r}_j -\mathbf{r}_k|) = g_{kj}(\mathbf{r}_k,\mathbf{r}_j)$. Note that the potential of mean force *w* is defined via $e^{-\beta w_{j,k}(\mathbf{r}_j,\mathbf{r}_k)}\equiv g_{jk}(\mathbf{r}_j, \mathbf{r}_k)$, implying

29 $$\begin{aligned} w_{j,k}\left( \mathbf{r}_j,\mathbf{r}_k\right) = w_{k,j}\left( \mathbf{r}_k,\mathbf{r}_j\right) . \end{aligned}$$

The reciprocity principle requires that the energy of the system be independent of the order in which it is assembled. Thus, one may bring ion *k* into position with ion *j* already fixed or vice versa. The left-hand side above is the work required to bring ion *j* into position with ion *k* already fixed, while the right-hand side corresponds to work required for bringing in ion *k* with ion *j* already fixed. Expressing this in terms of the separation and taking the negative gradient, one sees that this is also an average form of Newton’s third law.

The approximation from (25) to (26) thus implies the need of having

30 $$\begin{aligned} q_j<\phi (\mathbf{r}_j,\mathbf{r}_k)>^{(k)} = q_k <\phi (\mathbf{r}_k,\mathbf{r}_j)>^{(j)}\,, \end{aligned}$$

which is unlikely to hold when nonlinear terms are included. As Onsager stated, we have to expect the left-hand side differs from the right-hand side “except in very symmetric cases.” [3]

Interestingly, it is straightforward to verify that the solution the the Debye–Hückel equation satisfies this requirement. Moreover, this reciprocity has been explicitly proven [39] for the application of Debye–Hückel theory to an arbitrary collection of dielectric spheres in solution.

Another symmetry requirement arises in the form of a reciprocal relation. Since $U = (1/2) \sum _j q_j \sum _i q_i/(\epsilon _o |\mathbf{r}_i-\mathbf{r}_j|)$, it is easy to see that:

31 $$\begin{aligned} \frac{\partial F}{\partial q_j} = \phi _j \end{aligned}$$

where *F* is the Helmholtz free energy and $\phi _j$ is the average potential at $\mathbf{r}_j$ (excluding the contribution from $q_j$). Because the total differential of the electrostatic portion of the Helmholtz free energy is given by:

32 $$\begin{aligned} \mathrm {d}F = \sum _i \frac{\partial F}{\partial q_i} \mathrm {d}q_i = \sum _i \phi _i \mathrm {d}q_i, \end{aligned}$$

equality of the mixed partial derivatives implies:

33 $$\begin{aligned} \frac{\partial \phi _k}{\partial q_j} = \frac{\partial \phi _j}{\partial q_k} \end{aligned}$$

Importantly, the solution to the Debye–Hückel equation satisfies these requirements, but solutions to the Poisson–Boltzmann equation will not. Furthermore, it can be shown [5] that the Debye–Hückel equation is the exact small $\kappa$ limiting form of Eq. (24). Evidently, the Debye–Hückel equation has in some ways a firmer physical foundation than the Poisson–Boltzmann equation in spite of the fact that the former is obtained as a linearization of the latter. Moreover, as Onsager [3] points out, “as soon as the higher terms in the Poisson–Boltzmann equation become important, we can no longer expect the ionic atmospheres to be additive, and then the Poisson–Boltzmann equation itself becomes unreliable.”

A brief comment on van der Waals forces is perhaps appropriate at this point. The van der Waals interaction arises at the atomic scale as a result of quantum mechanical fluctuations. Thus, the ions in an ionic solution might well experience van der Waals interactions. However, while these forces are significant for atomic scale objects such as the ions in the solution, it will generally not be the case for the much larger colloidal objects (macroions), which deserve a classical approach. Nevertheless, this is not to say that there are no interactions of the form $r^{-6}$ among colloidal particles. An expansion in inverse powers of the separation *r* of the electrostatic interaction of polarizable objects in a polarizable medium will certainly include terms such as $r^{-6}$ [61], but their origin lies in continuum macroscopic electrostatics.

## Appendix B: Recapitulation of general formalism for arbitrary number of spheres

To make the presentation self-contained, let us provide here all of the definitions and equations [39] necessary for calculating the interaction energy for a system of arbitrary number of dielectric spheres immersed in an ionic fluid.

The total electrostatic energy of the system, *U*, is obtained via sums of weighted products of $\overline{Q}_{\ell m}$, the spherical components of free charge distributions, and $\breve{Q}^{+}_{\ell m}$, the spherical components of the net charge distributions that include charges induced on the surfaces of the spheres [39]:

34 $$\begin{aligned} U= & {} \frac{\kappa }{2} \sum _{k=1}^N \sum _{\ell m} \left[ i_\ell (\kappa a_k)\, k_\ell (\kappa a_k)\, \overline{Q}^{k\,*}_{\ell m}\, \breve{Q}^{k+}_{\ell m} - \frac{1}{ \epsilon _k \, \kappa a_k} \frac{\overline{Q}^{k*}_{\ell m} \, \overline{Q}^k_{\ell m} }{ (2\ell +1)} \right] \nonumber \\&+ \frac{\kappa }{2} \sum _{k=1}^N \sum _{j\ne k} \sum _{\ell m} \sum _{\ell _1 m_1}\, (-1)^{\ell _1}\nonumber \\&\quad \mathcal {H}_{\ell m \ell _1 m_1} (\kappa \mathbf {L}_{k\rightarrow j} )\, i_\ell (\kappa a_k)\, i_{\ell _1} (\kappa a_j)\, \overline{Q}^{k*}_{\ell m} \breve{Q}^{j+}_{\ell _1 m_1} . \end{aligned}$$

Here *k* (and *j*) enumerate the *N* spheres of radii $a_k$ ($a_j$) and dielectric constants $\epsilon _k$ ($\epsilon _j$), $\mathbf {L}_{k\rightarrow j}$ is a vector connecting the center of sphere *k* to that of sphere *j*, $\kappa$ is the inverse Debye length, $Y_{\ell m}(\hat{r})$ are the standard spherical harmonics with $\ell \in [0,\infty )$, $m \in [-\ell , \ell ]$, $i_\ell (z)$ are the modified spherical Bessel functions of the first kind with $i_\ell (z) = (i)^{-\ell } j_\ell (i\,z)$, while $k_\ell (z)$ are the modified spherical Bessel functions of the second kind with $k_\ell (z) = -(i)^{\ell } h^\mathrm{(1)}_\ell (i\,z)$.

The function $\mathcal {H}_{\ell m \ell _1 m_1} (\kappa \mathbf {L}_{k\rightarrow j} )$ takes care of different origins when expanding the surface charges around the center of each sphere:

35 $$\begin{aligned} \mathcal {H}_{\ell m \ell _1 m_1} \left( \kappa \mathbf {L}_{k\rightarrow j} \right) = \sum _{\ell _2 m_2} H^{\ell _1 m_1}_{\ell m \ell _2 m_2} k_{\ell _2}\left( \kappa L_{k\rightarrow j}\right) Y_{\ell _2 m_2} \left( \hat{L}_{k\rightarrow j}\right) , \nonumber \\ \end{aligned}$$

where

36 $$\begin{aligned} H^{\ell _1 m_1}_{\ell m \ell _2 m_2} \equiv C^{\ell _1\, 0}_{\ell \, 0\, \ell _2\, 0} \, C^{\ell _1 m_1}_{\ell \, m\, \ell _2 m_2} \sqrt{\frac{4\pi }{2\ell _1+1}} \sqrt{(2\ell +1)(2\ell _2+1)} \, , \nonumber \\ \end{aligned}$$

and $C^{\ell _1 m_1}_{\ell \, m\, \ell _2 m_2}$ are the Clebsch–Gordan coefficients. Note that $C^{\ell _1 0}_{\ell \, 0\, \ell _2 0} \ne 0$ only if $\ell + \ell _1 + \ell _2$ is even. Therefore, the sum in Eq. (35) should be taken only over $\ell _2$ of the same parity as $\ell +\ell _1$. This condition on the parity of the sum of the angular momenta leads to other useful relations such as $(-1)^{\ell _1} = (-1)^{\ell +\ell _2}$.

The spherical components $\overline{Q}^k_{\ell m}$ represent the free charge distribution on each sphere and are connected with the corresponding multipole moments $q_{\,\ell m}$ as:

37 $$\begin{aligned} \overline{Q}_{\ell m} \equiv \sqrt{4\pi }\, \frac{q_{\,\ell m}}{a^\ell } = \frac{\sqrt{4\pi }}{a^\ell } \int \rho (\mathbf {s}\,) \; s^\ell \, Y_{\ell m}^*(\hat{s}) \, d\mathbf {s}. \end{aligned}$$

These spherical components are surface equivalents of ordinary multipoles, and the rigorous equivalence of using either the ordinary multipoles or their surface equivalents has been demonstrated, both with ions [39] and without ions [54]. For convenience, the surface representation was used. In this case, the potential inside each sphere is a solution of the Laplace equation. The quantities $\breve{Q}^{k+}_{\ell m}$ are a scaled version of the spherical components of the net (free + induced) charge distributions, $\breve{Q}^{k}_{\ell m}$, that are solved for when there are no free ions in the dielectric medium [55]. The two sets of coefficients are connected via the following substitution rule:

38 $$\begin{aligned} \breve{Q}^{k+}_{\ell m} = \frac{\breve{Q}^k_{\ell m}}{\kappa a_k\, (2\ell +1)\, i_\ell (\kappa a_k)\, k_\ell (\kappa a_k)}. \end{aligned}$$

The spherical components of the scaled net charge $\breve{Q}^{k+}_{\ell m}$ are determined by the corresponding boundary conditions on the surfaces of the spheres that result in the following system of linear equations [39]:

39 $$\begin{aligned} \overline{Q}^k_{\ell m}= & {} i_\ell (\kappa a_k)\, k_\ell (\kappa a_k)\, \mathcal {K}(\ell , \kappa a_k) \, \breve{Q}^{k+}_{\ell m} \nonumber \\&+ \sum _{j\ne k} \sum _{\ell _1 m_1} (-1)^{\ell _1} \, i_\ell (\kappa a_k) \, i_{\ell _1}(\kappa a_j)\, \mathcal {I}(\ell , \kappa a_k) \nonumber \\&\quad \mathcal {H}_{\ell m \ell _1 m_1} (\kappa \mathbf {L}_{k\rightarrow j} ) \; \breve{Q}^{j+}_{\ell _1 m_1}, \end{aligned}$$

where, for the sake of compactness, we introduced the following notations:

40 $$\begin{aligned} \mathcal {K}\left( \ell , \kappa a_k\right)\equiv & {} \kappa a_k \left[ \ell \, \epsilon _k - \frac{\kappa a_k\, k'_\ell \left( \kappa a_k\right) }{ k_\ell \left( \kappa a_k\right) } \epsilon _o \right] , \quad \mathcal {I}\left( \ell , \kappa a_k\right) \nonumber \\\equiv & {} \kappa a_k \left[ \ell \, \epsilon _k - \frac{\kappa a_k \, i'_\ell \left( \kappa a_k\right) }{ i_\ell \left( \kappa a_k\right) } \epsilon _o \right] . \end{aligned}$$

One can avoid using derivatives of the modified spherical Bessel functions by employing the following equalities:

41 $$\begin{aligned} k'_\ell (x)=\frac{\ell \,k_\ell (x)}{x}-k_{\ell +1}(x), \quad i'_\ell (x)=\frac{\ell \,i_\ell (x)}{x}+i_{\ell +1}(x), \nonumber \\ \end{aligned}$$

so that

42 $$\begin{aligned} \mathcal {K}(\ell , \kappa a_k)= & {} \kappa a_k \left[ \ell (\epsilon _k -\epsilon _o) + \frac{\kappa a_k\, k_{\ell +1}(\kappa a_k)}{ k_\ell (\kappa a_k)} \epsilon _o \right] , \nonumber \\ \mathcal {I}(\ell , \kappa a_k)= & {} \kappa a_k \left[ \ell (\epsilon _k -\epsilon _o) - \frac{\kappa a_k \, i_{\ell +1}(\kappa a_k)}{ i_\ell (\kappa a_k)} \epsilon _o \right] . \end{aligned}$$

System (39), of course, is of infinite size, and the series in $\ell$ must be terminated at some $\ell _\mathrm{max}$, leaving $N (\ell _\mathrm{max}+1)^2$ equations. A significant advantage of the formalism [39] is that there is only one parameter, namely $\ell _\mathrm{max}$, that governs the accuracy of the obtained result. In contrast, termination of another expansion is necessary in the formalism of Ref. [49], while in Ref. [56], transposing the origin of spherical waves expansion is designed as a recursive procedure. Introducing such extra cut-offs complicates verification of the achieved accuracy.

An analysis of convergence of the electrostatic energy as a function of increasing $\ell _\mathrm{max}$ has been carried out in Ref. [39]. For two spheres of comparable sizes and charges, $\ell _\mathrm{max}= 10$ is sufficient for achieving relative error of $10^{-8}$ almost to the distance where the spheres touch (the so-called touching point). However, as we will see below, when there is variation in sphere radii or charge magnitude or placement, the value of $\ell _\mathrm{max}$ should be set higher.

In order to get the interaction energy, one has to subtract the sum of solvation energies of each sphere. The multipole components of the solvation energies are given [39] as:

43 $$\begin{aligned} U_\mathrm{solv} = \frac{\kappa }{2} \sum _{\ell m} \left[ \frac{2\ell +1}{\mathcal {K}(\ell , \kappa a)} - \frac{1}{\epsilon \, \kappa a} \right] \frac{\overline{Q}^*_{\ell m}\, \overline{Q}_{\ell m}}{ (2\ell +1)}. \end{aligned}$$

Thus,

44 $$\begin{aligned} U_\mathrm{int}= & {} \frac{\kappa }{2} \sum _{k=1}^N \sum _{\ell m} \left[ i_\ell (\kappa a_k)\, k_\ell (\kappa a_k)\, \overline{Q}^{k*}_{\ell m}\, \breve{Q}^{k+}_{\ell m} - \frac{\overline{Q}^{k*}_{\ell m} \, \overline{Q}^k_{\ell m} }{ \mathcal {K}(\ell , \kappa a_k)} \right] \nonumber \\&+ \frac{\kappa }{2} \sum _{k=1}^N \sum _{j\ne k} \sum _{\ell m} \sum _{\ell _1 m_1}\, (-1)^{\ell _1}\, \mathcal {H}_{\ell m \ell _1 m_1} \left( \kappa \mathbf {L}_{k\rightarrow j} \right) i_\ell \left( \kappa a_k\right) \, i_{\ell _1}\nonumber \\&\left( \kappa a_j\right) \, \overline{Q}^{k*}_{\ell m} \breve{Q}^{j+}_{\ell _1 m_1} . \end{aligned}$$

Finally, let us note that in the limit of $\kappa \rightarrow 0$ (no mobile ions in the solvent), the presented formalism reduces to the formalism of dielectric spheres in a dielectric medium [55], as shown in Ref. [39].

## Footnotes

1 Our implementation of the formalism [45] agrees with the formalism of papers [9, 39] only for $\ell =0$. Corrections from higher orders decrease much faster than they should, so, depending on parameters, the difference in the interaction energy can exceed a factor of 2. The situation with symmetric spheres, though, yields the minimum error, only few per cent. Finally, there is possibly a typo in their closed form for $\ell _\mathrm{max}=1$, as its numerical values do not fully match the corresponding values from their general formalism.

2 If one calculates not the error of the pairwise approximation in the *total* interaction energy, but rather its error in the *non-point-Coulomb* part of the total interaction energy (as was done in Ref. [61]), it is, in fact, of the order of 30%.
